# Spectrofluorometric and Molecular Docking Studies on the Binding of Curcumenol and Curcumenone to Human Serum Albumin

**DOI:** 10.3390/ijms16035180

**Published:** 2015-03-06

**Authors:** Omer Abdalla Ahmed Hamdi, Shevin Rizal Feroz, Jamil A. Shilpi, El Hassane Anouar, Abdul Kadir Mukarram, Saharuddin B. Mohamad, Saad Tayyab, Khalijah Awang

**Affiliations:** 1 Department of Chemistry, Faculty of Science, University of Malaya, 50603 Kuala Lumpur, Malaysia; E-Mail: omerhamdi2001@hotmail.com; 2 Institute of Biological Sciences, Faculty of Science, University of Malaya, 50603 Kuala Lumpur, Malaysia; E-Mails: shevrzl@gmail.com (S.R.F.); ak.mukarram@yahoo.com (A.K.M.); saharuddin@um.edu.my (S.B.M.); saadtayyab2004@um.edu.my (S.T.); 3 Center of Natural products and Drug Discovery (CENAR), University of Malaya, 50603 Kuala Lumpur, Malaysia; E-Mail: jamilshilpi@yahoo.com; 4 Atta-ur-Rahman Institute for Natural Product Discovery, Universiti Teknologi MARA, Kampus Puncak Alam, 42300 Bandar Puncak Alam, Malaysia; E-Mail: anouarelhassane@yahoo.fr

**Keywords:** *Curcuma zedoaria*, sesquiterpene, human serum albumin, protein-ligand interaction, molecular docking, fluorescence spectroscopy

## Abstract

Curcumenol and curcumenone are two major constituents of the plants of medicinally important genus of *Curcuma*, and often govern the pharmacological effect of these plant extracts. These two compounds, isolated from *C. zedoaria* rhizomes were studied for their binding to human serum albumin (HSA) using the fluorescence quench titration method. Molecular docking was also performed to get a more detailed insight into their interaction with HSA at the binding site. Additions of these sesquiterpenes to HSA produced significant fluorescence quenching and blue shifts in the emission spectra of HSA. Analysis of the fluorescence data pointed toward moderate binding affinity between the ligands and HSA, with curcumenone showing a relatively higher binding constant (2.46 × 10^5^ M^−1^) in comparison to curcumenol (1.97 × 10^4^ M^−1^). Cluster analyses revealed that site I is the preferred binding site for both molecules with a minimum binding energy of −6.77 kcal·mol^−1^. However, binding of these two molecules to site II cannot be ruled out as the binding energies were found to be −5.72 and −5.74 kcal·mol^−1^ for curcumenol and curcumenone, respectively. The interactions of both ligands with HSA involved hydrophobic interactions as well as hydrogen bonding.

## 1. Introduction

*Curcuma zedoaria* (Christm.) Rosc. (Zingiberaceae), also known as “white turmeric” is a perennial herb, largely found in tropical countries including Malaysia, Indonesia, India and Thailand [1]. In Malaysia, it is known as “temu putih” and is widely consumed as spice, flavoring agent for native dishes and an additive in food preparations for women in confinement after child birth [2,3]. It is extensively used in folk medicine for the treatment of menstrual disorders, dyspepsia, vomiting, and blood stagnation [1,4,5]. The plant is also used in the practice of traditional Chinese medicine for the treatment of cervical cancer [6].

**Figure 1:**
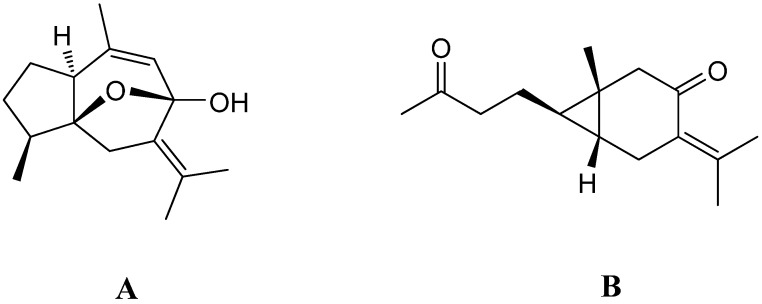
Chemical structures of curcumenol (**A**) and curcumenone (**B**).

The rhizomes of *C. zedoaria* are considered as a rich source of bioactive sesquiterpenes [7]. Curcumenol (**A**) and curcumenone (**B**) (Figure 1) are two of the most important sesquiterpenes possessing a number of beneficial biological activities. Curcumenol, a guaiane type sesquiterpene is known to exhibit analgesic [8], cytotoxic [9,10], hepatoprotective [11] and antimicrobial [12] properties, while curcumenone, a caraborane type sesquiterpene has been reported to be a vasorelaxant [13], hepatoprotective [11] and an effective inhibitor of intoxication [14].

Human serum albumin (HSA) serves as the primary transport protein in the human circulation capable of binding reversibly both endogenous and exogenous ligands such as fatty acids, hormones and drugs. It consists of 585 amino acids in a single polypeptide chain [15]. Crystallographic data has shown the presence of three homologous domains (I, II, and III), each comprised of subdomains A and B. The two high affinity ligand binding sites on HSA, known as Sudlow’s site I and II, are located in subdomains IIA and IIIA, respectively [16].

The interaction of a compound with serum albumin is known to influence its bioavailability, distribution, metabolism and elimination from the body. In particular, the affinity between a protein and a ligand affects the concentrations of the free and bound forms of the ligand as well as the duration of its half-life, which consequently determine its efficacy [15]. Therefore, investigations on the interaction of serum albumin with bioactive ligands may provide valuable information concerning their therapeutic efficacies [17].

Being the major and active constituents of *C. zedoaria*, curcumenol and curcumenone play important roles in exerting pharmacological activities after consumption of the herb. As the key components of a widely used natural product, curcumenol and curcumenone require the determination of pharmacokinetic parameters to establish their safety and efficacy. Furthermore, binding studies to HSA provide valuable parameters toward the establishment of the pharmacokinetic profile of such agents. In light of the above, the present report describes the binding properties of curcumenol and curcumenone to HSA based on fluorescence spectroscopic and molecular docking results.

## 2. Results and Discussion

### 2.1. Quenching Mechanism

**Figure 2:**
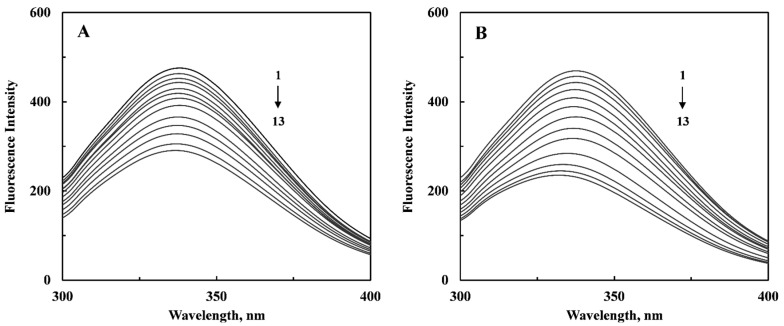
Emission spectra of human serum albumin (HSA) in the absence and the presence of increasing curcumenol (**A**) and curcumenone (**B**) concentrations, obtained in 20 mM sodium phosphate buffer, pH 7.4 upon excitation at 280 nm. [HSA] = 3 μM, [Ligand] = (1–13): 0, 3, 6, 9, 12, 15, 18, 24, 30, 37.5, 45, 52.5 and 60 μM. T = 25 °C.

Interactions between small ligands and macromolecules such as proteins have been widely investigated using fluorescence spectroscopy. Quenching of the protein fluorescence as well as shift in the emission maximum in the presence of a ligand can indicate a number of phenomena including complex formation, random collisions, energy transfer and excited state reactions [18,19,20]. Figure 2 shows intrinsic fluorescence spectra of HSA in the wavelength range, 300–400 nm upon excitation at 280 nm, obtained in the absence and the presence of increasing curcumenol and curcumenone concentrations. Addition of increasing concentrations of these ligands to HSA produced progressive decrease in the fluorescence intensity and significant blue shift in the emission maximum, suggesting interactions between these compounds and HSA. About 40% decrease in the fluorescence intensity at 338 and 2 nm blue shift were observed at the highest curcumenol concentration (60 μM), used in this study. On the other hand, curcumenone produced ~50% quenching in HSA fluorescence intensity, which was accompanied by a 6 nm blue shift in the emission maximum at the same ligand concentration. The shift in the emission maximum of HSA towards shorter wavelength suggested increased hydrophobicity in the microenvironment of the protein fluorophores upon interaction with these compounds [21]. It seems probable that ligand binding triggered the movement of hydrophobic residues in the vicinity of the fluorophores (Tyr and Trp), particularly around lone Trp-214, which is located in the vicinity of Sudlow’s site I [15]. It is important to note that Trp fluorescence makes major contribution in the protein fluorescence due to the presence of the emission maximum around 338 nm [21]. This has been supported by our docking results in which about half of the residues lining the binding pocket are purely hydrophobic in nature. Furthermore, other lining residues such as Lys (4 CH_2_ groups), Arg (3 CH_2_ groups), Tyr (benzene ring), Gln (2 CH_2_ groups) also contribute towards hydrophobicity of the binding pocket. Presence of hydrophobic milieu in the binding pocket makes favorable contribution in the ligand binding phenomenon as hydrogen bonds formed in the nonpolar environment seem to be much stronger.

**Figure 3:**
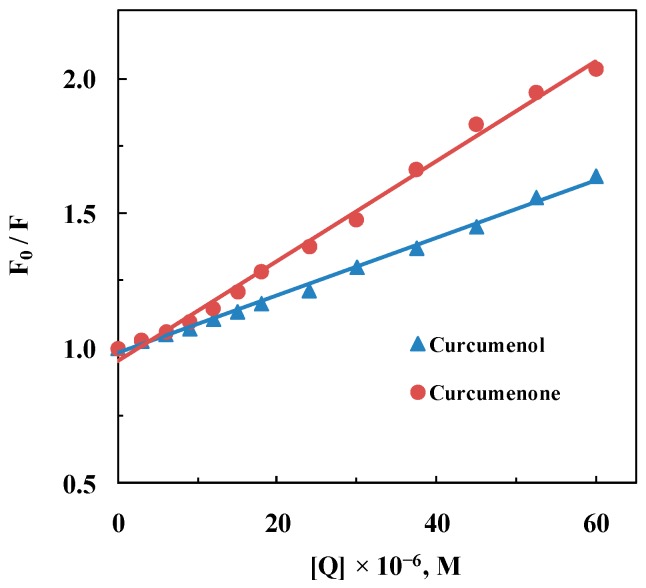
Stern-Volmer plots for the quenching of HSA fluorescence by curcumenol and curcumenone.

Although quenching of protein fluorescence can be regarded as an indication of binding between a compound and the protein, it is equally probable that the quenching phenomenon was due to collisions between the quencher molecule and the protein [21]. These two mechanisms of fluorescence quenching are known as static and collisional quenching, respectively, and can be differentiated by the bimolecular quenching constant, *k_q_* value, associated with the quenching process [21]. As a general rule, the *k_q_* value for a diffusion-controlled phenomenon typically falls in the region of 10^10^ M^−1^·s^−1^, while higher *k_q_* value indicates a binding reaction [21]. Figure 3 displays Stern-Volmer plots of the ligand–HSA systems, obtained after analyzing the quenching data using Equation (1). The resulting *K_SV_* and *k_q_* values for the interaction of both curcumenol and curcumenone with has are listed in Table 1. As can be seen, the *k_q_* values obtained were two orders of magnitude higher than the value expected for a process following the collisional quenching mechanism. This suggested that the quenching of HSA by curcumenol and curcumenone was governed by the static quenching mechanism, which involved the formation of a ground state complex between the ligands and HSA.

**Table 1:** Quenching and binding parameters for curcumenol-/curcumenone-HSA interactions, as obtained in 20 mM sodium phosphate buffer, pH 7.4 at 25 °C.

Parameter	Curcumenol	Curcumenone
*K_SV_*, M^−1^	1.07 × 10^4^	1.85 × 10^4^
*k_q_*, M^−1^·s^−1^	1.67 × 10^12^	2.90 × 10^12^
*K_b_*, M^−1^	1.97 × 10^4^	2.46 × 10^5^
*n*	1.07	1.26

### 2.2. Binding Parameters

**Figure 4:**
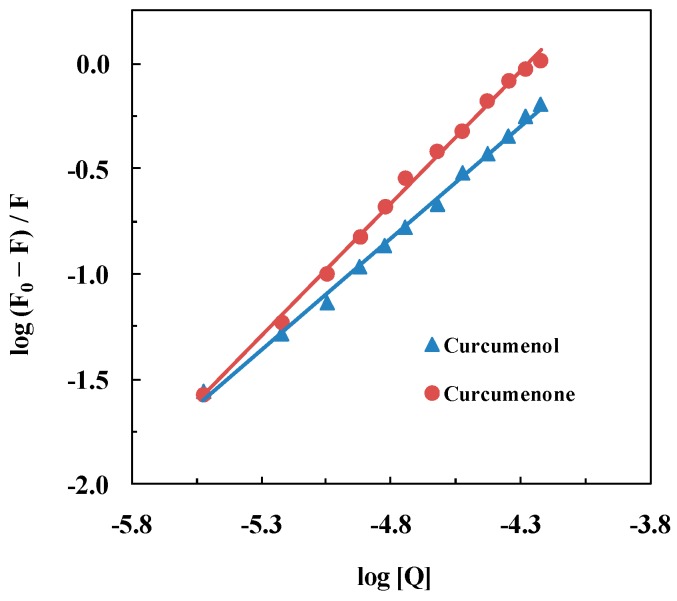
Double logarithmic plots for the interaction of HSA with curcumenol and curcumenone.

The binding constant (*K_b_*) for the ligand–HSA interaction and the number of binding sites (*n*) on the protein for these ligands were determined from the double logarithmic plots of log (*F_0_ − F*)/*F versus* log [*Q*] (Figure 4). The values of *n* and *K_b_* were obtained from the slope and the y-intercept of these plots, respectively, and are listed in Table 1. The values of *K_b_*, obtained for the interaction of curcumenol and curcumenone with HSA revealed intermediate affinity between these ligands and the protein. This was comparable to other ligand binding studies involving HSA [11,20,22,23]. It is worth noting that higher extent of quenching of HSA fluorescence by curcumenone was also reflected by its higher *K_b_* (2.46 × 10^5^ M^−1^) value in comparison to that obtained for the curcumenol–HSA system (1.97 × 10^4^ M^−1^). Interestingly, the value of *n* for the interaction of curcumenone with HSA was noticeably higher (1.26) compared to that (1.07) of curcumenol. There seems to be a higher likelihood of curcumenone to bind to other site(s) on the protein molecule in addition to its primary binding site. However, a slight difference in the value of “*n*” cannot be taken to clearly differentiate cooperative phenomenon with respect to curcumenone binding as double log Stern-Volmer equation suffers from several pitfalls.

### 2.3. Molecular Docking

**Figure 5:**
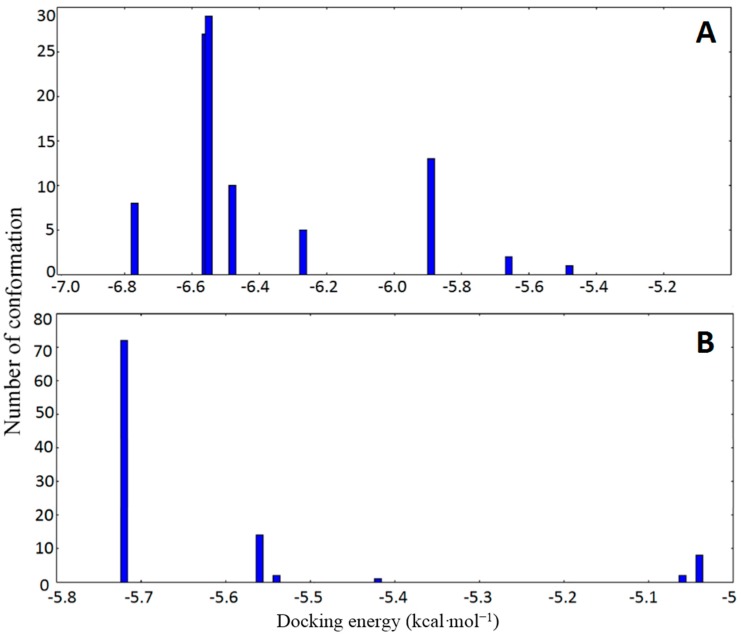
Cluster analyses of the AutoDock docking runs of curcumenol in the drug binding site I (**A**) and site II (**B**) of HSA (1BM0).

In order to predict the binding modes of curcumenol and curcumenone to the two main ligand binding sites of HSA (site I and site II), docking simulations of the interaction between these ligands and HSA were carried out. For each binding site, 100 docking simulations and clustering analysis at a root-mean-square deviation tolerance of 2.0 Å were performed. The docking analysis of 1BM0-curcumenol complex at the binding site I revealed a total of eight (8) multimember conformation clusters (Figure 5A) with the mean binding energy of −6.40 kcal·mol^−1^. The highest populated cluster had 29 out of 100 conformations. However, the configuration with the lowest binding energy (−6.77 kcal·mol^−1^) was not a member of the highest populated cluster. At the binding site II (Figure 5B), six (6) multimember conformation clusters, possessing mean binding energy of −5.53 kcal·mol^−1^ were identified, with the highest populated cluster having 72 members out of 100 conformations. Although the lowest binding energy configuration (−5.72 kcal·mol^−1^) was a member of the highest populated cluster, its binding energy was higher compared to the lowest binding energy configuration at site I (−6.77 kcal·mol^−1^). Thus, we predict that site I of HSA would be the preferable binding site of curcumenol to HSA (1BM0).

**Figure 6:**
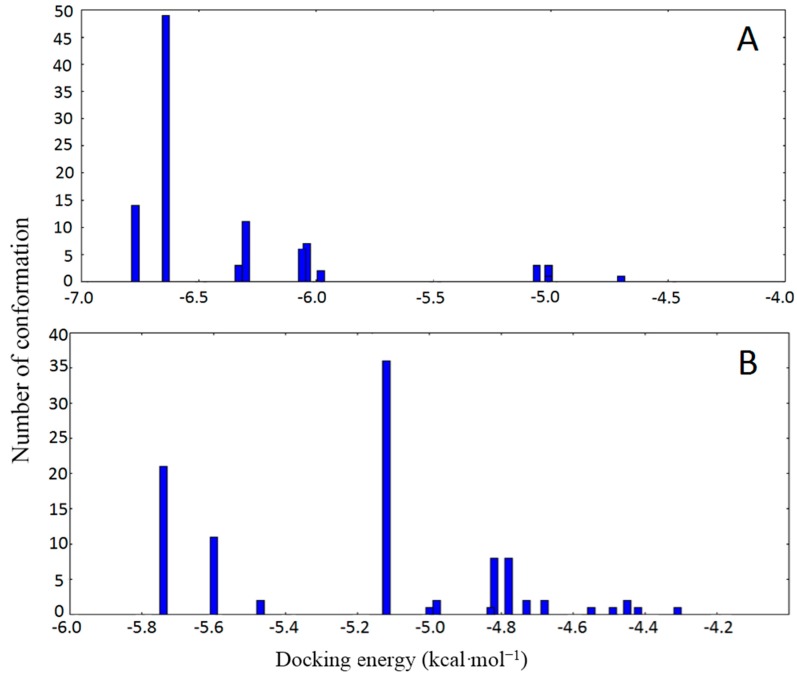
Cluster analyses of the AutoDock docking runs of curcumenone in the drug binding site I (**A**) and site II (**B**) of HSA (1BM0).

Clustering analysis of the 1BM0-curcumenone complex (Figure 6) revealed similar clustering pattern for both binding sites, as observed with the 1BM0-curcumenol system. The lowest binding energy conformation for site I of 1BM0-curcumenone was −6.77 kcal·mol^−1^, while that for site II was −5.74 kcal·mol^−1^. These observations also suggested the preference of site I for curcumenone binding to HSA.

**Figure 7:**
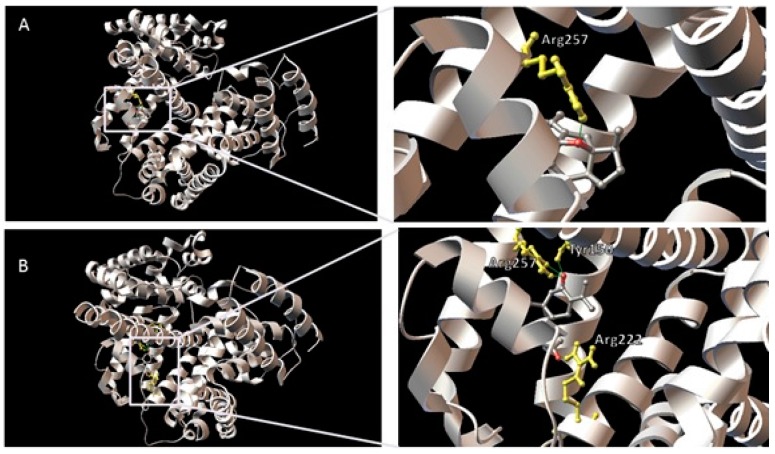
Predicted orientations of the lowest docking energy conformations of 1BM0-ligand complexes. The binding sites were enlarged to show hydrogen bonding (green lines) between amino acid residues and the ligands. Amino acid residues that form hydrogen bonds with the ligands are rendered in ball and stick and colored yellow. (**A**) Curcumenol in the binding site I of HSA (1BM0); (**B**) Curcumenone in the binding site I of HSA (1BM0).

**Figure 8:**
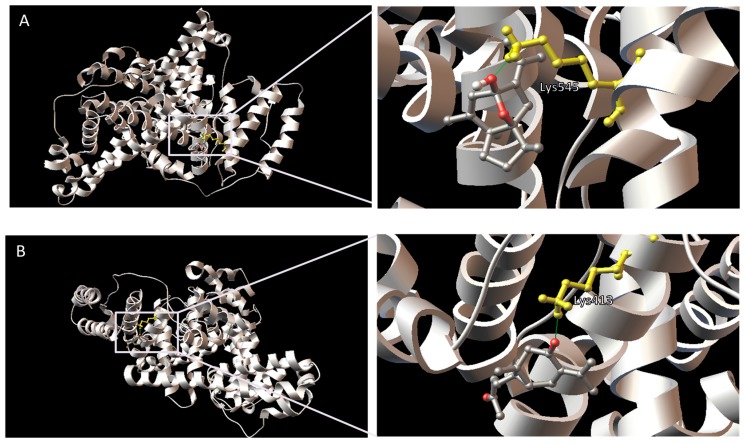
Predicted orientations of the lowest docking energy conformations of 1BM0-ligand complexes. The binding sites were enlarged to show hydrogen bonding (green lines) between amino acid residues and the ligands. Amino acid residues that form hydrogen bonds with the ligands are rendered in ball and stick and colored yellow. (**A**) Curcumenol in the binding site II of HSA (1BM0); (**B**) Curcumenone in the binding site II of HSA (1BM0).

The predicted binding models of curcumenol and curcumenone with the lowest docking energy (−6.77 kcal·mol^−1^) at site I of HSA were used for binding orientation analysis (Figure 7). Since the cluster analysis also supported the binding of curcumenol and curcumenone at site II of HSA, the lowest docking energy complex for curcumenol (−5.72 kcal·mol^−1^) and curcumenone (−5.74 kcal·mol^−1^) at site II of HSA were also analyzed (Figure 8). The ligand binding sites were defined as amino acid residues within 5 Å distance of the ligand. In the 1BM0-curcumenol complex at site I, the binding site was found to be located deep within the protein structure in a hydrophobic cleft walled by 18 amino acids: Tyr-150, Lys-195, Gln-196, Lys-199, Leu-219, Arg-222, Asp-237, Leu-238, Val-241, His-242, Arg-257, Leu-260, Ala-261, Lys-286, Ser-287, His-288, Ile-290 and Ala-291. However, in the 1BM0-curcumenone complex at the same site, the binding site was surrounded by 16 amino acids: Tyr-150, Lys-195, Gln-196, Lys-199, Leu-219, Arg-222, Phe-223, Leu-234, Leu-238, His-242,Arg-257, Leu-260 Ile-264, Ser-287, Ile-290 and Ala-291. The 1BM0-curcumenol complex formation at site II involved 14 amino acid residues, namely Leu-394, Leu-398, Lys-402, Phe-403, Asn-405, Ala-406, Val-409, Arg-410, Lys-413, Thr-540, Lys-541, Glu-542, Leu-544 and Lys-545. On the other hand, 1BM0-curcumenone complex at site II was associated with 13 amino acid residues: Leu-398, Lys-402, Phe-403, Asn-405, Ala-406, Leu-407, Val-409, Arg-410, Lys-413, Thr-540, Lys-541, Glu-542, and Lys-545.

Presence of hydrophobic amino acid residues at the binding sites of HSA might have contributed towards the stability of the ligand-HSA complex through hydrophobic interactions. However, the presence of several polar amino acid residues within the proximity of the bound ligands indicated that the interactions between these ligands and HSA at both site I and site II cannot be presumed to be exclusively hydrophobic. Furthermore, in the 1BM0-curcumenol complex docking conformation, one hydrogen bond was predicted involving the hydrogen atom of Arg-257 and the ethereal oxygen atom of curcumenol (Table 2). For the 1BM0-curcumenone complex docking conformation, 4 hydrogen bonds were predicted involving hydrogen atoms from three different amino acid residues of HSA (Tyr-150, Arg-222 and Arg-257) and the oxygen atoms of the acetyl and ketone groups of curcumenone. In contrast, there was only one hydrogen bond in the 1BM0-curcumenone complex at site II, formed by the oxygen atom of curcumenone and the hydrogen atom of Lys-413 (Table 2). Both ligands showed one hydrogen bonding at the binding site. For 1BM0-curcumenol complex at site II, the hydrogen bond formed between the hydrogen atom of Lys-545 and the oxygen atom of hydroxyl group while for 1BM0-curcumenone complex, the hydrogen bond was associated with the hydrogen atom of Lys-413 and oxygen of the ketone group (Table 2).

**Table 2:** Predicted hydrogen bonds between interacting atoms of the amino acid residues of HSA (1BM0) and the ligands at site I and site II.

Compound	HSA Binding Site	Protein Atom	Ligand Atom	Distance (Å)
Curcumenol	Site I	Arg257:HH22	O (ethereal)	1.907
	Site II	Lys545:HZ3	O (hydroxyl)	1.684
Curcumenone	Site I	Tyr150:HH	O (ketone)	1.907
		Arg222:HH11	O (acetyl)	1.930
		Arg257:HE	O (ketone)	1.969
		Arg257:HH22	O (ketone)	2.236
	Site II	Lys413:HZ3	O (ketone)	2.025

These results suggested that although curcumenol and curcumenone can bind to both sites I and II of HSA, the binding is more preferable at site I. Although, the lowest binding energies for both ligands at site I were the same (−6.77 kcal·mol^−1^) in molecular docking study, the 1BM0-curcumenone complex involved a greater number of hydrogen bonding (four) than that of 1BM0-curcumenol complex (one). This is reflected in the fluorescence quenching assay where curcumenone showed a higher extent of fluorescence quenching compared to curcumenol. Thus, it can be inferred that both curcumenol and curcumenone showed similar HSA binding properties, with curcumenone having a slightly higher affinity towards HSA.

## 3. Experimental Section 

### 3.1. Materials

Human serum albumin (HSA), essentially fatty acid free was obtained from Sigma-Aldrich Inc. (St. Louis, MO, USA). All other chemicals were of analytical grade purity.

### 3.2. Isolation and Purification of Curcumenol and Curcumenone

The rhizomes of *C. zedoaria* (1.0 kg) were finely powdered and macerated with *n*-hexane for three days at room temperature, followed by successive extractions with dichloromethane and ethyl acetate, and finally with Soxhlet extraction using methanol. The *n*-hexane extract yielded 24.2 g (2.4%), out of which 20.0 g were subjected to silica gel column chromatography. The elution was performed initially using *n*-hexane followed by *n*-hexane/ethyl acetate gradient. Fractions were then combined according to similarity of the TLC spots to give 21 fractions. Fraction 8 was further purified using preparative thin layer chromatography to give curcumenol (15.5 mg), whereas curcumenone (16.4 mg) was purified from fractions 12 and 13. The structures were established through extensive spectroscopic studies and were found consistent with previous reports [24,25].

### 3.3. Preparation of Protein and Ligand Solutions

The stock solution of HSA was prepared in 20 mM sodium phosphate buffer, pH 7.4 and its concentration was determined spectrophotometrically using a specific extinction coefficient value of 5.3 at 280 nm [26]. Stock solutions of curcumenol and curcumenone were prepared by dissolving desired quantities of their crystals in ethanol and diluting to the desired concentrations with 20 mM sodium phosphate buffer, pH 7.4.

### 3.4. Ligand Binding Studies

The interactions of curcumenol and curcumenone with HSA were studied using fluorescence quench titration method [23,27]. The concentration of HSA was maintained at 3 μM, while the ligand concentrations ranged from 0–60 μM. The final volume of the reaction mixture was made up to 3 mL with 20 mM sodium phosphate buffer, pH 7.4. The samples were allowed to equilibrate for 30 min prior to fluorescence measurements. The fluorescence spectra of these samples were recorded at 25 °C on a Jasco FP-6500 spectrofluorometer with a 1 cm path length quartz cuvette. Both excitation and emission slits were set at 10 nm, while the scan speed was maintained at 500 nm·min^−1^. The samples were excited at 280 nm, and the emission spectra were recorded in the wavelength range, 300–400 nm.

### 3.5. Analysis of the Binding Data

In order to reveal the quenching mechanism of HSA fluorescence, the binding data were analyzed according to the Stern-Volmer equation [21]:

(1) $$F_{0}/F=\;K_{SV}\left[Q\right]+1=\;k_{q}\tau_{0}\left[Q\right]+1$$

where *F*_0_ and *F* are the fluorescence intensities of HSA in the absence and the presence of the quencher, respectively, *K_SV_* is the Stern-Volmer constant, [Q] is the quencher concentration, *k_q_* is the bimolecular quenching constant and τ_0_ is the fluorescence lifetime of free HSA. A value of 6.38 × 10^−9^ s was used for τ_0_ [28].

The binding constant, *K_b_* and the number of binding sites, *n* for ligand–HSA interactions were determined by treating the fluorescence data according to the following equation (Equation (2)) [18]:

(2) $$\text{log}\left(F_{0}-F\right)/F=n\text{log}\left[Q\right]+\text{log}K_{b}$$

### 3.6. Molecular Modelling

The structures of curcumenol and curcumenone were drawn using ACD/ChemSketch Freeware (Advanced Chemistry Development Inc., Toronto, ON, Canada), 3-D optimized and exported as a mol file. The geometry optimization of these structures was refined with the VegaZZ 2.08 [29] batch processing MOPAC script (mopac.r; keywords: MMOK, PRECISE, GEO-OK) using AM1 semiempirical theory [30] and converted and stored as a mol2 file. Molecular docking, visualization and rendering simulation were performed using AutoDock 4.2 [31] and AutoDockTools 1.5.4 [32] at the Academic Grid Malaysia Infrastructure. For the docking study, the curcumenol and curcumenone non-polar hydrogens were merged and the rotatable bonds were defined. The crystal structure of HSA (PDB code 1BM0, res. 2.5 Å) was downloaded from the Protein Data Bank (PDB) [33]. Its water molecules were removed and the atomic coordinates of chain A were stored in a separate file and used as input for AutoDockTools, where polar hydrogens, Kollman charges and solvation parameters were added. The two binding sites (site I and site II) were defined using two grids of 70 × 70 × 70 points each with a grid space of 0.375 Å centered at coordinates *x* = 35.26 *y* = 32.41 *z* = 36.46 for site I and *x* = 14.42 *y* = 23.55 *z* = 23.31 for site II. Lamarckian genetic algorithm with local search (GA-LS) was used as the search engine, with a total of 100 runs for each binding site. In each run, a population of 150 individuals with 27,000 generations and 250,000 energy evaluations were employed. Operator weights for crossover, mutation and elitism were set to 0.8, 0.02 and 1, respectively. For local search default parameters were used. Cluster analysis was performed on docked results using RMS tolerance of 2.0 Å. The protein-ligand complexes were visualized and analyzed using AutoDockTools (The Scripps Research Institute, La Jolla, CA, USA).

## 4. Conclusions

Curcumenol and curcumenone are well known as plant-derived natural products finding their use in therapeutic applications in recent years. The present investigation provides an insight into the HSA binding properties of these pharmacologically important molecules. Curcumenol and curcumenone showed similar binding characteristics, with the latter having a stronger affinity to HSA. Both compounds bind to site I and site II on HSA, facilitated by hydrophobic forces and hydrogen bonds. The data presented can be useful in the establishment of their pharmacokinetic profiles in the process of future drug development.
